# Re-innervation of the Denervated Dentate Gyrus by Sprouting Associational and Commissural Mossy Cell Axons in Organotypic Tissue Cultures of Entorhinal Cortex and Hippocampus

**DOI:** 10.3389/fnmol.2019.00270

**Published:** 2019-11-12

**Authors:** Domenico Del Turco, Mandy H. Paul, Viktor J. Beeg Moreno, Lars Hildebrandt-Einfeldt, Thomas Deller

**Affiliations:** Institute of Clinical Neuroanatomy, Dr. Senckenberg Anatomy, Neuroscience Center, Goethe University Frankfurt, Frankfurt, Germany

**Keywords:** calretinin, calretinin-knock out, collateral sprouting, entorhinal cortex lesion, perforant path transection, plasticity, regeneration

## Abstract

Collateral sprouting of surviving axons contributes to the synaptic reorganization after brain injury. To study this clinically relevant phenomenon, we used complex organotypic tissue cultures of mouse entorhinal cortex (EC) and hippocampus (H). Single EC-H cultures were generated to analyze associational sprouting, and double EC-H cultures were used to evaluate commissural sprouting of mossy cells in the dentate gyrus (DG) following entorhinal denervation. Entorhinal denervation (transection of the perforant path) was performed at 14 days *in vitro* (DIV) and associational/commissural sprouting was assessed at 28 DIV. First, associational sprouting was studied in genetically hybrid EC-H cultures of beta-actin-GFPtg and wild-type mice. Using calretinin as a marker, associational axons were found to re-innervate almost the entire entorhinal target zone. Denervation experiments performed with EC-H cultures of Thy1-YFPtg mice, in which mossy cells are YFP-positive, confirmed that the overwhelming majority of sprouting associational calretinin-positive axons are mossy cell axons. Second, we analyzed associational/commissural sprouting by combining wild-type EC-H cultures with calretinin-deficient EC-H cultures. In these cultures, only wild-type mossy cells contain calretinin, and associational and commissural mossy cell collaterals can be distinguished using calretinin as a marker. Nearly the entire DG entorhinal target zone was re-innervated by sprouting of associational and commissural mossy cell axons. Finally, viral labeling of newly formed associational/commissural axons revealed a rapid post-lesional sprouting response. These findings demonstrate extensive and rapid re-innervation of the denervated DG outer molecular layer by associational and commissural mossy cell axons, similar to what has been reported to occur in juvenile rodent DG *in vivo*.

## Introduction

Regardless of the underlying cause, central nervous system (CNS) injuries result in primary damage at the lesion site and secondary denervation damage in connected brain areas. Thus, even spatially circumscribed CNS lesions cannot be considered local injuries since every lesion challenges the neuronal network. In line with this view of CNS damage, the CNS reacts to lesions with a profound rewiring of its connections. This lesion-induced plastic response may homeostatically restabilize or destabilize the perturbed network, causing changes in the throughput of information in denervated areas (Steward, 1994; Vlachos et al., 2012a, b, 2013). In some cases, the reorganization of surviving pathways may even be functionally restorative, resulting in the recovery of some of the functions that have been lost (Fawcett, 2009; Garcia-Alias et al., 2009; Maier et al., 2009; Filli and Schwab, 2015).

One of the mechanisms contributing to the reorganization of connections in denervated areas of the CNS is collateral sprouting of surviving axons. Although collateral sprouting was described several decades ago, and considerable efforts have gone into understanding the rules of sprouting (Steward, 1994; Deller and Frotscher, 1997; Deller et al., 2007; Perederiy and Westbrook, 2013), we do not fully understand the dynamics, specificity, or the regulatory mechanisms underlying axon sprouting, needed to exploit its potential in therapy. Although rehabilitation of patients with CNS injuries aims at training and enhancing the function of surviving pathways and, thus, the rewiring mechanisms, there are no standardized pharmacological treatments that target sprouting in a clinical setting (Filli and Schwab, 2015). Thus, a reinvestigation of the mechanisms underlying denervation-induced collateral sprouting may be useful in the search for novel targets.

A useful strategy for addressing the above questions related to sprouting is the use of an *in vitro* denervation model in which sprouting conditions can be controlled. Accordingly, we transferred the classic *in vivo* entorhinal denervation model (Steward et al., 1974; Lynch et al., 1976; Cotman and Nadler, 1978; Steward, 1994; Deller and Frotscher, 1997) to organotypic tissue cultures, and demonstrated robust collateral sprouting of associational axons following entorhinal denervation *in vitro* (Prang et al., 2003; Del Turco and Deller, 2007). Using this same model, we analyzed dendritic changes of granule cells in response to entorhinal denervation and identified sphingosin-1-phosphate (S1P) as a regulatory molecule and potential therapeutic target (Willems et al., 2016). Furthermore, we demonstrated the role of homeostatic plasticity mechanisms in the context of brain reorganization (Vlachos et al., 2012a, b, 2013). In all of these studies, single entorhinal cortex (EC) and hippocampus (H) cultures (EC-H) were used in which associational mossy cell axons sprout and re-innervate the entorhinal zone (Prang et al., 2003). Under *in vivo* conditions, however, mossy cells have commissural collaterals, which contribute to the re-innervation of the contralateral dentate gyrus *in vivo* (Del Turco et al., 2003). Whether these collaterals would also sprout *in vitro* has remained unclear. To study associational and commissural mossy cell sprouting systematically, and to extend the model for sprouting analysis, we have built complex tissue culture systems using double cultures of EC and H. In these complex cultures, we revisited associational sprouting and studied, for the first time, commissural sprouting *in vitro*. Our data indicate that both collaterals of the mossy cells sprout and contribute to the re-innervation of the denervated entorhinal target zone in the dentate outer molecular layer. Mossy cell sprouting *in vitro* is extensive and similar to what has been reported previously in juvenile rodents (Gall and Lynch, 1978, 1980; Gall et al., 1980; McWilliams and Lynch, 1983).

## Materials and Methods

### Animals

Thy1-YFPtg (Feng et al., 2000) and beta-actin-GFPtg (Okabe et al., 1997) reporter mice, as well as calretinin-deficient (Schurmans et al., 1997) and wild-type mice (C57Bl6/J), were used in this study. Mice were maintained in a 12 h light/dark cycle with food and water available *ad libitum*. Every effort was made to minimize distress of animals. Animal care and experimental procedures were performed in agreement with the German law on the use of laboratory animals (animal welfare act; TierSchG). Animal welfare was supervised and approved by the Institutional Animal Welfare Officer of Goethe-University Frankfurt, Faculty of Medicine.

### Organotypic Tissue Cultures

Entorhino-hippocampal tissue cultures were prepared from mouse brain at postnatal days 4–5 according to previously published protocols (Prang et al., 2003; Del Turco and Deller, 2007). To prepare complex cultures of wild-type hippocampus with GFP-positive entorhinal cortex, animals were decapitated and wild-type hippocampi without attached entorhinal cortex and entorhinal cortex (EC) of beta-actin–GFPtg hippocampi were dissected. For Thy1-YFPtg cultures, animals were decapitated and hippocampi with attached entorhinal cortex (entorhino-hippocampal culture, EC-H) were dissected. To prepare complex cultures of two entorhino-hippocampal cultures, animals were decapitated and EC-H of wild-type and calretinin-deficient animals were dissected. For brain dissection, ice-cold preparation medium (Minimal essential medium (MEM, Gibco) containing 2 mM Glutamax (Gibco), pH 7.3) was used. Slices (300–350 μm) were cut using a Leica vibratome (VT1200S, Leica). Organotypic tissue cultures were maintained on porous-membrane filter insets (Millicell-CM, Millipore) and incubated in a humidified atmosphere with 5% CO_2_ at 37°C. Medium for cultivation contained 42% MEM, 25% basal medium eagle (Gibco), 25% heat-inactivated normal horse serum (Gibco), 2.5% HEPES buffer solution (Invitrogen), 0.15% bicarbonate (Invitrogen), 0.675% glucose (Sigma-Aldrich), 0.1 mg/ml streptomycin (Sigma-Aldrich), 100 U/ml penicillin (Sigma-Aldrich) and 2 mM Glutamax. The pH was adjusted to 7.3 and the medium was replaced every 2–3 days. Organotypic tissue cultures were incubated for up to 28 days *in vitro* (DIV).

### Perforant Path Lesion *in vitr*o

Entorhino-hippocampal cultures were allowed to mature until 14 DIV. Using a sterile scalpel blade, entorhino-hippocampal cultures were completely transected as described (Del Turco and Deller, 2007). After the lesion, the cultures were placed back into the incubator and kept until 14 days post lesion.

### Adeno-Associated Virus Production

HEK293T cells were transfected with pDP1rs (Plasmid Factory), pDG (Plasmid Factory), and GFP-vector plasmid (12:8:5) by calcium phosphate seeding and precipitation adapted from Grimm (2002). Cells were collected 48 h after transfection, washed twice with Phosphate Buffered Saline (PBS), centrifuged at 800 rpm for 4 min and re-suspended in PBS. Viral particles within the cells were released by 4 freeze- and thaw cycles and supernatant was centrifuged at 10,000 rpm for 10 min to remove cell debris. The final supernatant was collected, aliquoted, and stored at −80 °C.

### Viral Labeling

To label hilar mossy cells, tissue cultures were transduced with an adeno-associated virus serotype 2 (AAV2) containing GFP under the human Synapsin 1 promoter (AAV2-hSyn1-GFP). Injections were performed using an injection pipette pulled from thin-walled borosilicate capillaries (Harvard Apparatus, 30-0066). Pipettes were held by a head stage with a HL-U holder (Axon Instruments) and positioned using a micro-manipulator (Luigs and Neumann). Approximately 0.05–0.1 μl of AAV2-hSyn1-GFP was injected directly into the hilar region of the dentate gyrus using a syringe. Tissue cultures were visualized with an upright microscope (Nikon FN1) using a 10× water immersion objective lens (Nikon Plan Fluor, NA 0.30). All injections were performed 3–4 days after the tissue cultures were prepared.

### Time-Lapse Imaging of Organotypic Tissue Cultures

Live imaging of tissue cultures was performed as previously described (Muller et al., 2010). The membrane insert with the cultures was placed into a 30 mm petri dish that contained imaging medium (37 °C) which consisted of NaCl 129 mM, KCl 4 mM, MgCl_2_ 1 mM, CaCl_2_ 2 mM, glucose 4.2 mM, HEPES 10 mM, Trolox 0.1 mM, streptomycin 0.1 mg/ml, penicillin 100 U/ml; pH 7.4. The osmolarity of the imaging medium was adjusted with sucrose to the osmolarity of the incubation medium. Imaging was done with an upright confocal microscope (Zeiss, Pascal; 488 nm excitation laser) equipped with a temperature-regulated stage (37 °C), using a 10× water immersion objective lens (0.3 NA; Zeiss) to visualize tissue cultures and to identify AAV-labeled mossy cells in the hilus of the DG. Image stacks (approximately 20 images per stack; *z*-axis interval between consecutive frames: 5 μm) of DG regions were obtained at a resolution of 1024 × 1024 pixels. Tissue cultures were imaged on 14, 17, 20, 24, and 28 days *in vitro* for less than 10 min per culture in order to keep exposure time and phototoxic damage minimal. GFP-intensity levels of AAV-injected cultures were equalized at DIV 14 using Adobe Photoshop CS6 (Version 13.0.1 × 64) to account for differences in GFP expression between cultures.

### Immunofluorescence

Tissue cultures were fixed in 4% paraformaldehyde (PFA) in 0.1 M PBS (pH 7.4) for 4 h. After several washes with 50 mM Tris–buffered saline (TBS; pH 7.4), cultures were re-sliced into 30 μm sections on a vibratome (VT 1000S, Leica). For cutting on a cryostat cultures were fixed in 4% PFA in 0.1 M PBS (pH 7.4) and 4% sucrose for 1 h at room temperature (RT), followed by 2% PFA and 30% sucrose in PBS overnight at 4°C. After several washes with 50 mM TBS (pH 7.4), cultures were re-sliced into 30 μm sections on a cryostat (CM3050 S, Leica). Free-floating sections were washed several times in 50 mM TBS containing 0.1% Triton X-100, incubated in a blocking buffer (0.5% Triton X-100, 5% bovine serum albumin (BSA) in 50 mM TBS) for 30 min at RT and subsequently incubated with the appropriate primary antibody (diluted in 0.1% Triton X-100, 1% BSA in 0.05 M TBS) for 2 days at RT. Rabbit anti-calretinin (1:1000, Swant) and mouse anti-NeuN (1:1000, Chemicon) were used as primary antibodies. After several washing steps, sections were incubated with the appropriate secondary Alexa-conjugated antibodies (1:2000, Invitrogen) for 4 h at RT, counterstained with DRAQ5 (1:10,000, Thermo Fisher Scientific) or Hoechst (1:50,000, Sigma-Aldrich) to visualize nuclei, and mounted in Fluorescence Mounting Medium (Dako, Agilent Technologies).

### Quantification of the Sprouting Response Following Denervation

To quantify the expansion of the associational or commissural fiber plexus from the inner into the denervated outer molecular layer, confocal image stacks (EZ-C1, Nikon) of middle sections from re-sliced tissue cultures were captured (10× objective; pinhole size: 30 μm). Using the counterstains to visualize the cytoarchitecture of the DG, the width of the total molecular layer was determined from the outer border of the granule cell layer to the hippocampal fissure using ImageJ (Schneider et al., 2012). This was done at three locations (0°, −60°, + 60°), with 0° being defined as a line from the middle of the hilus through the crest of the DG to the hippocampal fissure. In a second step, the width of the calretinin-positive or the YFP-positive fiber plexus was measured from the outer border of the granule cell layer to the outer border of the main plexus. The percentage of the molecular layer covered by the calretinin-positive or the YFP-positive fiber plexus was calculated for the three locations in control and denervated cultures and subsequently averaged per culture. This algorithm ensured defined sampling of the supra- and infrapyramidal blades of the DG as well as the crest region and, thus, yielded an overall parameter of the extent of sprouting.

### Statistics

Statistical analysis was done using GraphPad Prism 6 Software. Mann–Whitney test or Kruskal–Wallis test was used. Significance level was set at: ^∗^*p* < 0.05. Individual tests and test parameters are indicated in figure captions.

## Results

### Laminar Termination of Calretinin- Positive Associational and Entorhinal Axons in the Dentate Gyrus of Re-sliced Entorhino-Hippocampal Hybrid Cultures

**FIGURE 1:**
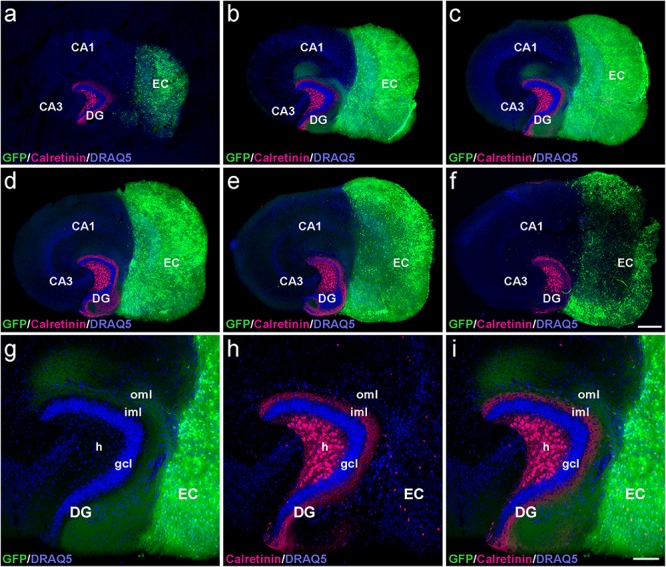
Serial confocal sections of re-sliced entorhino-hippocampal culture. **(a–f)** Serial sections (30 μm) of a control entorhino-hippocampal tissue culture (DIV 28) composed of wild-type mouse hippocampus and beta-actin-GFPtg mouse entorhinal cortex (EC). Note the laminar termination pattern of calretinin-positive axons (magenta) in the inner molecular layer (iml) and of entorhinal axons (GFP; green) in the outer molecular layer (oml) of the dentate gyrus (DG). Although fiber lamination is maintained throughout all levels of the re-sliced DG **(a–f)**, the best-defined and most organotypic termination pattern of the two projections is seen in middle sections **(b,c)**. Accordingly, such sections were used for the quantification of denervation-induced axonal sprouting. **(g–i)** Higher magnification of the middle section illustrated in **(c)**, exhibiting a well-organized DG with an organotypic cyto- and fiber-architecture. DRAQ5 (blue) was employed to visualize nuclei. h, hilus; gcl, granule cell layer; CA1, cornu ammonis subfield 1; CA3, cornu ammonis subfield 3. Scale bars: 250 μm **(a–f)**; 100 μm **(g–i)**.

To determine whether the laminar termination pattern of the two major projection systems to the dentate gyrus, i.e., the calretinin-positive associational projection arising from hilar mossy cells and the entorhinal projection arising from layer II neurons of the entorhinal cortex (EC), were maintained in organotypic tissue cultures, genetically hybrid cultures were generated at postnatal day 4–5. Entorhinal cortex derived from a beta-actin-GFPtg reporter mouse was co-cultured with hippocampus obtained from wild-type mice, and maintained *in vitro* until DIV 28. After fixation, re-slicing and immunolabeling for calretinin, essentially all calretinin-positive mossy cell axons and all GFP-positive entorhinal axons present within the culture could be visualized using confocal imaging (Figure 1). Although a segregated termination of calretinin-positive associational and entorhinal axons could be seen at all levels of the re-sliced DG (Figure 1), the most defined termination pattern of the two projections was found in middle sections (Figures 1b,c). At this culture level, the shape of the granule cell layer as well as the laminar termination pattern of calretinin-positive fibers in the inner molecular layer and entorhinal axons in the outer molecular layer appeared closest to the *in vivo* situation, i.e., most organotypic (Figures 1g–i).

In the part of our study in which we analyzed and quantified denervation-induced sprouting, we focused on the middle sections of re-sliced cultures. We consider this methodological approach important, since it takes the three-dimensional organization of the tissue cultures into account and clear tissue borders are needed for reliable quantification of the sprouting response. In addition, the shape of the granule cell layer varied toward more superficial levels, possibly because proliferation and differentiation of granule cells continues *in vitro* for days after preparation of the culture (Radic et al., 2017).

### Associational Sprouting of Mossy Cell Axons Following Lesion

**FIGURE 2:**
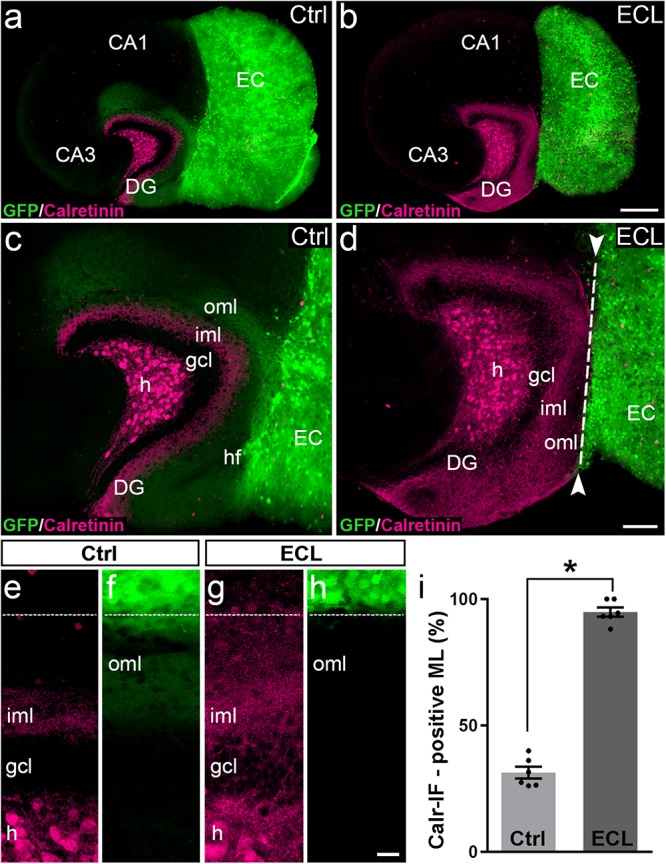
Associational sprouting of calretinin-positive axons following lesion at DIV 14. **(a)** Control (Ctrl) entorhino-hippocampal culture (re-sliced; middle section) composed of wild-type mouse hippocampus and beta-actin-GFPtg mouse entorhinal cortex (EC) at DIV 28. Mossy cell axons (calretinin-immunolabeling; magenta) and entorhinal axons (green) terminate in their respective layers of the dentate gyrus (DG). **(b)** Following transection of the entorhinal projection at DIV 14, EC axons are lost and an extensive sprouting of calretinin-positive mossy cells axons into the “entorhinal zone” of the DG is observed 14 days post lesion (DIV 28). **(c–h)** Higher magnifications of the cultures shown in **(a)** and **(b)**. In the non-lesioned culture **(c,e,f)** calretinin-positive fibers occupy the inner molecular layer (iml). In the lesioned culture **(d,g,h)** calretinin-positive fibers cover the iml and the outer molecular layer (oml) up to the hippocampal fissure (hf; dashed line). **(i)** Quantitative analysis of the sprouting response of calretinin-positive axons. The width of the main calretinin plexus in the molecular layer (ML) was measured in control (31.32 ± 2.33%, Ctrl) and EC-lesioned cultures (94.82 ± 1.87%, ECL). Mann–Whitney test; mean ± SEM; ^∗^*p* < 0.05; *N* = 6 cultures per group; h, hilus; gcl, granule cell layer; CA1, cornu ammonis subfield 1; CA3, cornu ammonis subfield 3. Scale bars: 250 μm **(a,b)**; 100 μm **(c,d)**; 20 μm **(e–f)**.

After assessing the 3D-organization, we used the hybrid EC-H tissue cultures described above for the analysis of associational sprouting following entorhinal denervation (Figure 2). At DIV 14 the entorhino-hippocampal projection was transected using a sterile scalpel and at DIV 28 associational sprouting was analyzed in middle sections of re-sliced cultures labeled for calretinin (Figures 2b,d). Non-lesioned cultures (DIV 28) served as controls (Figures 2a,c). Because GFP-positive entorhinal axons could be identified using direct fluorescence, completeness of the lesion could be verified by the absence of EC axons in the dentate gyrus prior to re-slicing of the culture.

In controls (Figures 2a,c,e,f) the calretinin-positive fiber plexus in the inner molecular layer was well-defined, easy to identify, and it covered ∼30% of the width of the molecular layer (Figure 2i). Of note, in addition to the calretinin-positive mossy cells, some hippocampal interneurons also contain calretinin (Blasco-Ibanez and Freund, 1997). The axons of these interneurons are present throughout the culture, including the molecular layer of the dentate gyrus. However, compared to the dense calretinin-positive plexus in the inner molecular layer formed by hilar mossy cells, calretinin-positive axons of presumed interneurons are scarce. The border between the calretinin-positive mossy cell axons and the “entorhinal zone” of the molecular layer is, thus, well-defined and amenable to quantification (Figures 2a,c,e).

Following entorhinal denervation (Figures 2b,d,g,h) GFP-positive EC axons were lost. In contrast, virtually the entire molecular layer was filled with calretinin-positive axons. These axons reached up to the hippocampal fissure and covered ∼95% of the width of the molecular layer (Figure 2i). This result was highly reproducible, robust and massive, since the density of calretinin-positive fibers in the denervated zone was close to the density of calretinin-positive fibers in the inner molecular layer in some cases (e.g., Figures 2d,g).

We conclude from our observations *in vitro* that sprouting calretinin-positive axons extensively re-innervate the entire “entorhinal zone” of the slice-cultured dentate gyrus.

### Changes in Calretinin- Immunofluorescence Observed After Entorhinal Denervation Reflect Sprouting of Hilar Mossy Cell Axons

**FIGURE 3:**
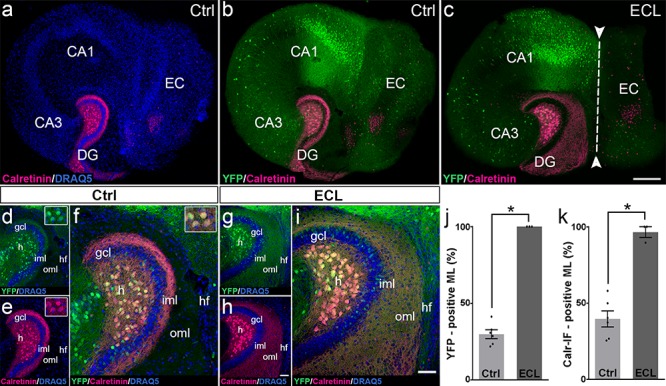
Changes in calretinin-immunofluorescence observed after entorhinal denervation reflect sprouting of hilar mossy cell axons. **(a–i)** Entorhino-hippocampal tissue cultures (DIV 28) generated from Thy1-YFPtg mouse hippocampus immunolabeled for calretinin (magenta) and counterstained with DRAQ5 (blue). Overviews **(a,b)** and higher magnifications **(d–f)** of the dentate gyrus (DG) of a non-denervated control culture reveal YFP- (green) and calretinin- (magenta) positive axons in the inner molecular layer (iml) of the DG. Note the almost complete overlap of the two markers **(f)**. Insets show YFP- and calretinin-positive mossy cell somata in the hilus (h). Overview **(c)** and higher magnification **(g–i)** of the DG of a culture with an entorhinal cortex lesion (ECL; dashed line in c indicates knife cut) performed at DIV 14. YFP-positive mossy cell axons **(g)** as well as calretinin-positive axons **(h)** are abundant in the denervated outer molecular layer (oml) and reach up to the hippocampal fissure (hf). Note almost complete overlap of the two markers. **(j,k)** Quantitative analysis of the axonal sprouting response into the molecular layer (ML). Comparable results are obtained for YFP (29.81 ± 2.87%, Ctrl; 100 ± 0%, ECL) and calretinin (Calr; 39.65 ± 5.22%, Ctrl; 96.53 ± 3.47%, ECL). Mann–Whitney test; mean ± SEM; ^∗^*p* < 0.05; *N* = 3–6 cultures per group; gcl, granule cell layer; CA1, cornu ammonis subfield 1; CA3, cornu ammonis subfield 3. Scale bars: 200 μm **(a–c)**; 50 μm **(d–i)**.

The re-innervation of the outer dentate molecular layer by calretinin-immunopositive axons strongly suggests that mossy cell axons leave their classical “associational” termination zone in the inner molecular layer and invade the “entorhinal zone” of the dentate gyrus. However, since calretinin is also present in the axons of some GABAergic interneurons (Blasco-Ibanez and Freund, 1997), which may also sprout following entorhinal denervation (Nadler et al., 1974; Nadler, 1981; Goldowitz et al., 1982; Deller et al., 1995a; Houser, 2014), we wanted to corroborate and validate our findings using a second approach. To accomplish, we generated EC-H cultures of Thy1-YFP-transgenic animals (Feng et al., 2000). In the dentate gyrus of these cultures, variable numbers of granule cells and mossy cells express the YFP-transgene (Figure 3). In some cultures, mossy cells predominate, and a well-defined YFP-positive mossy cell axon plexus was observed in the inner molecular layer (Figures 3b,d). Both, mossy cell somata and mossy cell axons targeting the inner molecular layer were strongly calretinin-positive (Figures 3a,b,d–f) in these YFPtg culture preparations.

Next, we performed an entorhinal denervation experiment and denervated EC-H cultures prepared from Thy1-YFPtg mouse brains at DIV 14. At DIV 28, the cultures were fixed, re-sliced, and immunolabeled for calretinin. Similar to what we have shown above for calretinin immunolabeling (Figure 2), calretinin-immunopositive axons were abundant throughout the denervated “entorhinal target zone” (Figures 3h,i). This pattern of sprouting was mirrored by YFP-positive fibers arising from hilar mossy cells (Figures 3g,i). Following denervation, calretinin and YFP-positive fibers covered 96% and 100% of the width of the molecular layer, respectively (Figures 3j,k). The overlay of both markers indicates that the overwhelming majority of axons re-innervating the denervated outer molecular layer of the dentate gyrus are double-labeled, i.e., are mossy cell axons.

We conclude from these experiments that associational mossy cell axons are primarily responsible for the re-innervation of the denervated dentate gyrus in EC-H single cultures (denervation occurring at DIV 14).

### Associational and Commissural Sprouting Following Bilateral Entorhinal Denervation of Wild-Type and Calretinin-Deficient Double Cultures

In the intact brain, axons of hilar mossy cells form both the ipsilateral inner molecular layer (the associational projection) and the same region of the contralateral dentate gyrus (the commissural projection). Therefore, we determined whether the commissural axon collateral of mossy cells *in vitro* forms the same reorganization as it does *in vivo*.

**FIGURE 4:**
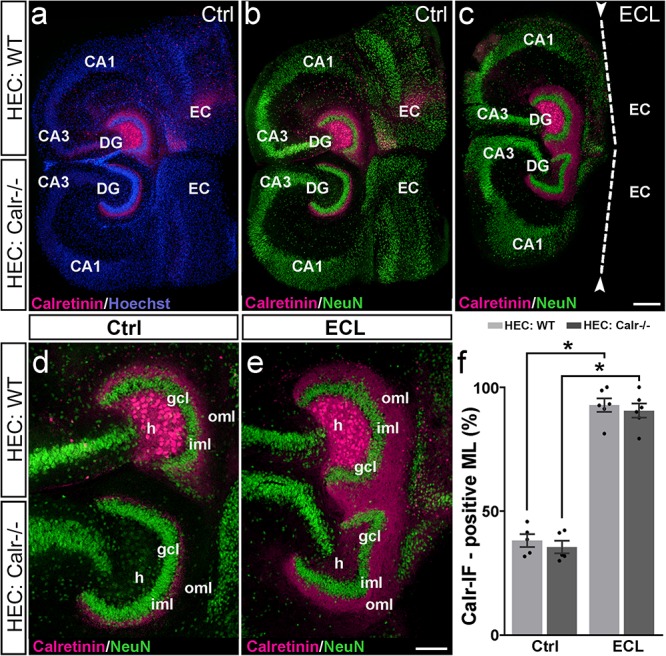
Associational and commissural sprouting following bilateral entorhinal denervation of wild-type and calretinin-deficient double cultures. **(a–c)** Hybrid double cultures of wild-type (WT) and calretinin-deficient (Calr−/−) mouse hippocampus (DIV 28) immunolabeled for calretinin (magenta) and counterstained with Hoechst (blue) or NeuN (green). Overview **(a,b)** and higher magnification **(d)** of a non-denervated double culture. Calretinin-positive mossy cells found only in the hilus (h) of the wild-type culture form associational (wild-type culture) and commissural (Calr−/− culture) collaterals to the inner molecular layer (iml). Overview **(c)** and higher magnification **(e)** of a double culture in which the entorhinal projection has been removed at DIV 14 (entorhinal cortex lesion, ECL; dashed line in **(c)** indicates knife cut). At DIV 28 calretinin-positive axons are abundant in the outer molecular layer (oml) of the dentate gyrus (DG) on both the ipsi- (wild-type; associational) and contralateral (Calr−/−; commissural) side. **(f)** Quantitative analysis of the axonal sprouting response into the molecular layer (ML). On the wild-type side (associational sprouting; light gray bars) a significant expansion of the calretinin-plexus occurs (38.16 ± 2.59%, Ctrl; 92.84 ± 2.72%, ECL). On the Calr−/− side (commissural sprouting; dark gray bars) a similar expansion of the calretinin-plexus is detected (35.56 ± 2.57%, Ctrl; 90.61 ± 2.86%, ECL). Kruskal-Wallis with Dunn’s multiple comparison *post hoc* test; mean ± SEM; ^∗^*p* < 0.05; *N* = 5–6 cultures per group; gcl, granule cell layer; CA1, cornu ammonis subfield 1, CA3, cornu ammonis subfield 3; EC, entorhinal cortex. Scale bars: 200 μm **(a–c)**; 100 μm **(d–e)**.

One way to address this question is to study the response of both axon collaterals to entorhinal denervation *in vitro*. Since calretinin is a robust marker for mossy cells and their axons, we used calretinin to study sprouting of both mossy cell axon collaterals. To distinguish between ipsi- and contralateral mossy cell axons, we co-cultured an EC-H culture generated from the brain of a wild-type mouse (mossy cells are calretinin-positive), with an EC-H culture generated from the brain of a calretinin-deficient mouse in which the mossy cells are calretinin-negative. Immunostaining of these cultures for calretinin only reveals the calretinin-positive wild-type axons whereas the calretinin-negative mossy cell axons arising from the calretinin-deficient EC-H culture are unlabeled (Figure 4).

Indeed, in calretinin-immunolabeled control double cultures, calretinin-positive mossy cell somata were seen only in the hilus of the wild-type culture (Figure 4). In line with this, the associational and commissural calretinin-positive axon plexuses were present in the ipsi- and contralateral dentate gyrus (Figures 4a,b,d), covering 38 and 36% of the molecular layer, respectively (Figure 4f). We then performed an entorhinal cortex lesion at DIV 14 and quantified the expansion of both calretinin-positive fiber plexus at DIV 28. At this time point, calretinin-positive fibers covered nearly the entire molecular layer in both dentate gyri (Figures 4c,e,f). Calretinin-positive axons covered 93 and 91% of the width of the molecular layers associationally and commissurally, respectively (Figure 4f).

We conclude from our findings that entorhinal denervation can induce associational as well as commissural mossy cell sprouting.

### Associational and Commissural Sprouting Begins Within Days After Entorhinal Denervation of Wild-Type Double Cultures

**FIGURE 5:**
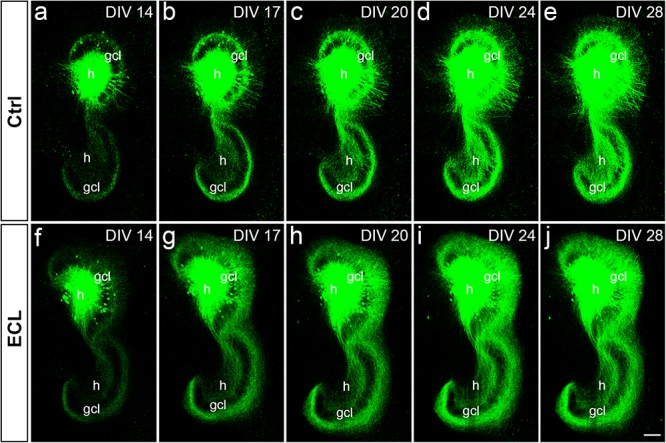
Associational and commissural sprouting in wild-type double cultures after entorhinal denervation visualized by hilar injection of AAV2-hSyn1-GFP. **(a–j)** Time-lapse imaging of wild-type entorhino-hippocampal double cultures without **(a–e)** and with entorhinal cortex lesion [ECL; **(f–j)**]. At DIV 4 all cultures received an injection of AAV2-hSyn1-GFP into one hilus (h). On DIV 14 one group of cultures received bilateral ECL **(f–j)**, whereas the other group served as non-lesioned control **(a–e)**. Both groups were treated identically and imaged (whole mount cultures) with the same imaging parameters at the time points indicated until DIV 28. Although the expression level of GFP (green) increased in both groups with observation time, the termination pattern of GFP-positive associational and commissural fibers showed major differences: Whereas GFP-positive associational and commissural fibers were most abundant in the inner molecular layer (iml) of the non-lesioned culture, GFP-associational and commissural fibers also invaded the outer molecular layer (oml) of the ECL culture. Sprouting into the oml occurred as already as 3 days post lesion [**(g)**; DIV 17] and seemed to be complete around 10 days post lesion [**(i)**; DIV 24]. gcl, granule cell layer; Scale bar: 100 μm.

**FIGURE 6:**
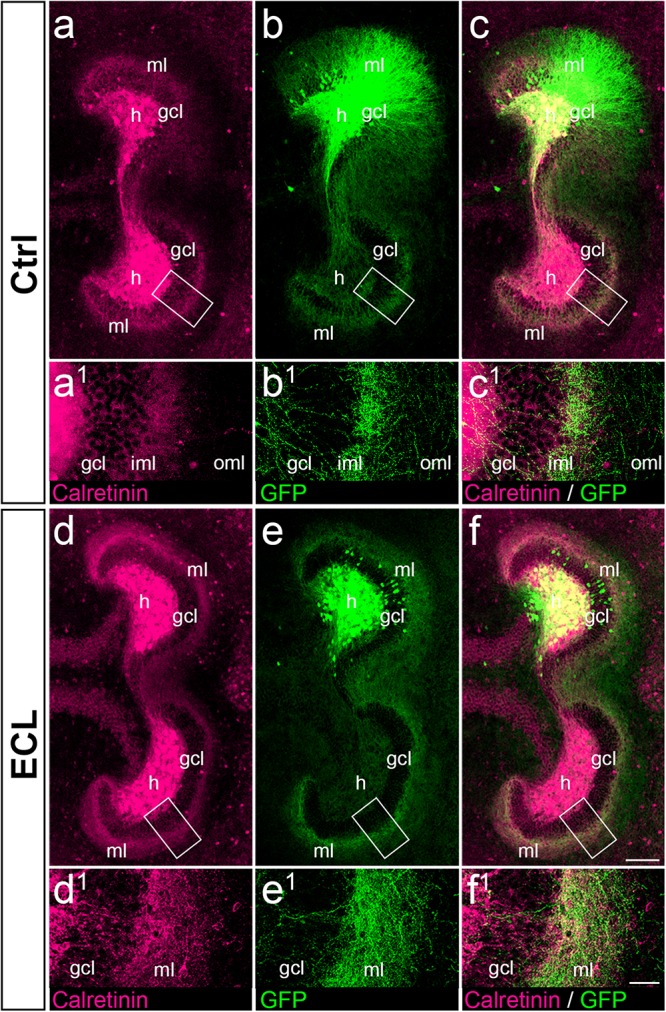
Associational and commissural sprouting after entorhinal denervation of AAV2-hSyn1-GFP injected wild-type double cultures corresponds to the sprouting of calretinin-positive fibers. **(a–c)** Entorhino-hippocampal double culture (DIV 28; control (Ctrl) culture; fixed and re-sliced) immunolabeled for calretinin (magenta) and injected with AAV2-hSyn1-GFP (green; injection at DIV 4). Overlay of **(a)** and **(b)** shown in **(c)**. Boxed areas are illustrated at higher magnification **(a^1^–c^1^)**. Note the preference of associational/commissural calretinin-positive and of commissural GFP-positive axons for the inner molecular layer (iml). **(d–f)** Entorhino-hippocampal double culture with entorhinal cortex lesion (ECL; 14 days post lesion; DIV 28; fixed and re-sliced). Overlay of **(d)** and **(e)** shown in **(f)**. Boxed areas are illustrated at higher magnification **(d^1^–f^1^)**. Calretinin-positive associational/commissural axons as well as GFP-positive commissural axons are found throughout the entire molecular layer and show extensive overlap. Scale bars: 100 μm **(a–f)**; 20 μm **(a^1^–f^1^)**.

Next, we assessed the dynamics of sprouting after *in vitro* entorhinal cortex lesion. To address this question, wild-type EC-H double cultures were prepared and AAV2-hSyn1-GFP was injected into one hilus. At 14 DIV, one group of cultures was lesioned (Figures 5f–j), whereas the other group served as non-lesioned controls (Figures 5a–e). Otherwise, both groups were treated identically and repetitively imaged as whole mounts (Figure 5). At the end of the imaging period some cultures were fixed, re-sliced, and immunostained for calretinin (Figure 6; see next paragraph).

In both groups, the AAV successfully transduced granule cells and hilar neurons with hSyn1-GFP. In general, injections were limited to the DG. Within the injected dentate gyrus, the site and size of the injection, as well as the number of transduced cells varied between cultures. Expression of GFP was detected ∼8–10 days following injection of the virus and GFP expression levels increased throughout the entire observation period (Figure 5). Because of this change in GFP expression we used only differences in the distribution pattern (and not in signal strength) of GFP-labeled axons between time-matched control and lesioned cultures for the evaluation of sprouting.

Control cultures injected with AAV showed in the molecular layer ipsilateral to the injection (“associational side”) a dense GFP-labeled axon plexus in the inner molecular layer and numerous GFP-labeled axons in the outer molecular layer. In those injections in which some granule cells had also been transduced, GFP-labeled granule cell dendrites were present in parts of the molecular layer. Of note, the border of the GFP-axon plexus in the inner molecular layer, which arises from mossy cell axons, could be well-delineated. Although the intensity of the GFP-signal increased with time, the termination pattern of these inner molecular layer fibers remained apparently unchanged in controls. Likewise, in the molecular layer of the dentate gyrus contralateral to the injection (“commissural side”) a dense GFP-labeled axon plexus was seen in the inner molecular layer. A much smaller number of GFP-labeled commissural axons were also found in the outer molecular layer, as seen *in vivo* (Deller et al., 1995b, 1996). Similar to the associational side, the outer border of the commissural GFP-plexus in the inner molecular layer could be readily defined. In controls, the termination pattern of these commissural fibers remained apparently unchanged.

In denervated cultures injected with the AAV, the situation was similar to controls at DIV 14. However, as early as DIV 17, GFP-positive fibers from the inner molecular layer were observed to enter the denervated “entorhinal target zone” of the dentate gyrus. This sprouting response continued and appeared to be relatively complete by DIV 24, i.e., 10 days post lesion. We conclude from these data that associational/commissural sprouting starts rapidly, i.e., within only a few days following entorhinal denervation *in vitro* at DIV 14.

### Associational and Commissural Sprouting Seen in AAV2-hSyn1- GFP-Injected Wild-Type Double Cultures Mirrors Sprouting of Calretinin-Positive Fibers

Since AAV injections transduce all types of neurons, we wanted to confirm that the majority of GFP-positive axons seen in the inner molecular layer of controls and in the entire molecular layer of denervated cultures were calretinin-positive. As shown *in vivo* (Blasco-Ibanez and Freund, 1997), and in this *in vitro* study (Figure 3), calretinin primarily labels mossy cell axons and is a marker for mossy cell axon sprouting. Accordingly, we fixed, re-sliced, and immunostained the AAV-injected wild-type double cultures for calretinin (Figure 6).

In control as well as in denervated cultures an extensive overlap between GFP-positive and calretinin-positive axons was seen (Figure 6), in particular in the inner molecular layer of controls (Figures 6a–c) and in the entire molecular layer following denervation (Figures 6d–f). As expected, overlap of the two markers was not as complete as that seen in the case of the YFPtg cultures (c.f. Figure 3), since some GFP-positive axons do not arise from mossy cells.

## Discussion

In the absence of any specific treatment for CNS damage, e.g., stem cells to replace neurons, or drugs that might induce axonal regeneration to restore anatomical connectivity, lesion-induced reorganization in the CNS might be an effective strategy. Since collateral sprouting is one of the mechanisms in this rewiring process, it appears to be timely to reinvestigate its rules and mechanisms using *in vitro* systems, such as the entorhinal denervation model *in vitro* (Prang et al., 2003; Del Turco and Deller, 2007; Vlachos et al., 2012a, 2013).

**FIGURE 7:**
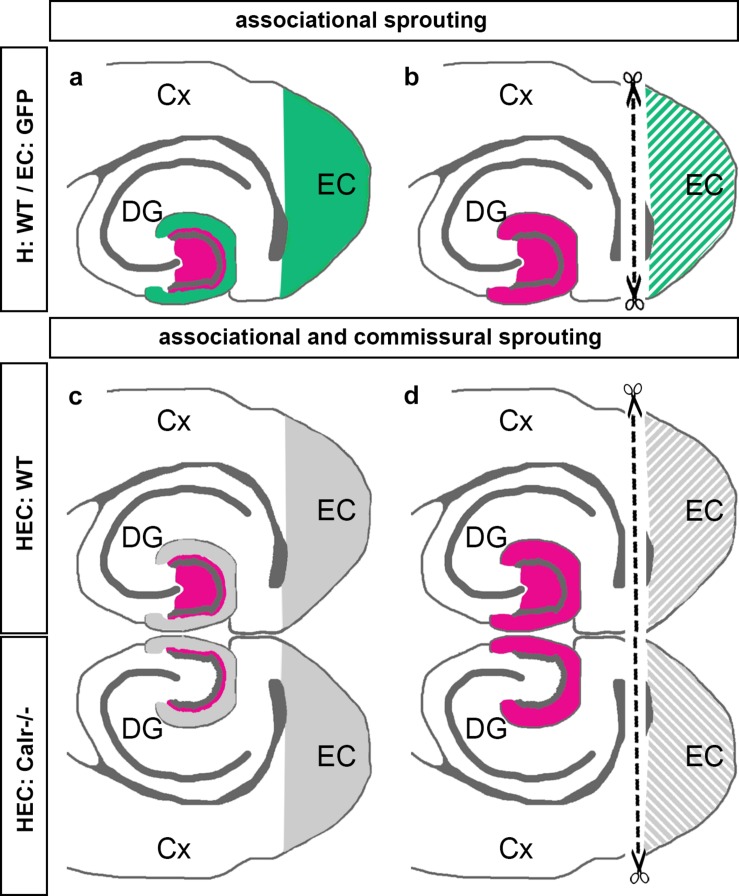
Schematic summary diagram of associational and commissural sprouting following entorhinal denervation in organotypic tissue cultures of mouse hippocampus. **(a,b)** Associational sprouting following entorhinal denervation at DIV 14 in single entorhino-hippocampal tissue cultures (c.f. Figures 1–3). **(a)** In non-denervated control cultures hilar mossy cells (magenta) terminate in the inner molecular layer of the dentate gyrus (DG). Entorhinal axons (green) terminate in the outer molecular layer. **(b)** Following transection and loss of entorhino-hippocampal axons (dashed line) associational mossy cell axons sprout and cover the “entorhinal zone” of the DG. **(c,d)** Associational and commissural sprouting following denervation at DIV 14 in double entorhino-hippocampal tissue cultures generated from wild-type (HEC: WT) and calretinin-deficient (HEC: Calr−/−) mouse hippocampus (c.f. Figure 4). **(c)** In non-denervated double cultures calretinin-positive mossy cells are only found in the HEC: WT culture. Axons of these neurons (magenta) innervate the ipsilateral (associational) and the contralateral (commissural) inner molecular layer of the DG. Using this hybrid approach associational and commissural axons of mossy cells can be identified using calretinin-immunofluorescence. Entorhinal fibers are shown in gray. **(d)** Following entorhinal cortex lesion (dashed line) associational and commissural calretinin-positive mossy cell axons sprout into the outer molecular layer, i.e., the “entorhinal zone” of the DG, on both sides; Cx, cortex; EC, entorhinal cortex.

The present study was performed to better understand the sprouting of mossy cell axons following entorhinal denervation *in vitro*. Our findings can be summarized as follows: (1) Using EC-H co-cultures of beta-actin-GFPtg and wild-type mice, we could show that calretinin-positive associational axons re-innervate the denervated “entorhinal” zone of the DG (Figures 7a,b). (2) Using EC-H cultures of Thy1-YFPtg mice, we demonstrated that calretinin-immunolabeling can be used to visualize mossy cell sprouting. (3) In double cultures of EC-H, a hippocampal commissural system forms. By co-culturing EC-H of wild-type and EC-H of calretinin-deficient mice, the associational and commissural collaterals of the wild-type mossy cell population could be distinguished. Following entorhinal lesions of these double cultures, re-innervation of the entorhinal zone by associational axons was seen in the wild-type DG and re-innervation by commissural axons was observed on the calretinin-deficient side (Figures 7c,d). (4) Using AAV injected wild-type double-cultures, we found that associational/commissural sprouting of mossy cell axons occurs rapidly post-lesion. Taken together, we conclude that the associational and the commissural collaterals of mossy cells respond rapidly to entorhinal denervation *in vitro*. The speed and extent of the *in vitro* sprouting response was similar to that reported in juvenile rodents *in vivo*.

### Calretinin as a Marker for the Analysis of Sprouting Mossy Cell Axons

In previous work (Prang et al., 2003), we provided the first evidence for the sprouting of associational calretinin-positive axons after entorhinal denervation *in vitro*. In our present study, we revisited this phenomenon using genetically hybrid and re-sliced EC-H co-cultures. The data show that sprouting of associational calretinin-positive axons is robust, with sprouting calretinin-positive axons re-innervating the molecular layer all the way to the hippocampal fissure.

The use of calretinin as a marker for mossy cell axons is possible in mouse tissue, because mossy cells in mice, in contrast to rat mossy cells, express this calcium-binding protein constitutively (Blasco-Ibanez and Freund, 1997). However, calretinin is not only present in mossy cells, but also in a subpopulation of interneurons in the DG and hippocampus (Blasco-Ibanez and Freund, 1997). Since interneurons have been shown to contribute to the re-innervation of the denervated DG (Nadler et al., 1974; Nadler, 1981; Goldowitz et al., 1982; Deller et al., 1995a), we could not exclude that a substantial fraction of sprouting calretinin-positive fibers arises from such calretinin-positive interneurons. Therefore, we decided to use a second approach and employed EC-H cultures generated from Thy1-YFPtg mouse brain. In these cultures, mossy cells and granule cells but not calretinin-positive interneurons express YFP and, thus, sprouting YFP-positive axons in the molecular layer following entorhinal denervation are sprouting mossy cell axons. Using these cultures, we were able to directly compare mossy cell axon sprouting to the sprouting response seen with calretinin immunolabeling, which reflects sprouting of calretinin-positive mossy cells and, potentially, sprouting of calretinin-positive interneurons. This approach also allowed us to detect and estimate the fraction of sprouting axons arising from calretinin-positive interneurons. Of note, almost all YFP-positive axons found in the denervated outer molecular layer were also calretinin-positive (and vice versa), demonstrating that the overwhelming majority of calretinin-positive axons re-innervating the molecular layer after entorhinal denervation originate from mossy cell axons. Compared to the number of sprouting mossy cell axons, sprouting of axons of calretinin-positive interneurons appears to be, in fact, negligible. We also conclude, that calretinin is an excellent and highly robust marker that can be used to visualize sprouting mossy cell axons in mouse tissue cultures.

However, if Thy1-YFPtg tissue cultures are more specific for mossy cell axons, why did we continue to use calretinin-immunolabeling to visualize the sprouting response instead of switching to Thy1-YFPtg cultures? Several methodological reasons explain this choice: In our hands the use of calretinin-immunofluorescence is technically simple, results are highly robust and the extent of sprouting can readily be quantified. Also, the standardized use of calretinin as a marker for sprouting mossy cell axons makes it possible to compare the extent of sprouting across all conditions. In contrast, preparations from Thy1-YFPtg mice contain variable numbers of YFP-positive mossy and granule cells, considerably increasing the number of cultures needed for experimentation. Only a minority of cultures show a useful YFP-expression pattern. This is an even greater constraint in the case of YFP-positive double-cultures. Thus, although Thy1-YFPtg cultures were of the essence to demonstrate the overlap of calretinin and mossy cells, they do not appear to be the ideal choice for the routine analysis of sprouting, e.g., in high-throughput assays.

### Calretinin-Deficient Mice as a Tool to Dissect Associational and Commissural Mossy Cell Sprouting

After validating calretinin as an excellent marker for sprouting mossy cell axons, we considered analyzing mossy cell sprouting in double cultures of wild-type EC-H. In such double cultures organotypic commissural projections exist and mossy cells project axon collaterals into the ipsi- as well as the contralateral DG. However, within the inner molecular layer of the DG, calretinin-positive associational collaterals from one side mix with calretinin-positive commissural collaterals from the other side. Since calretinin-immunolabeling will visualize both collaterals, this does not allow for a separate analysis of the two mossy cell axon collaterals. To solve this problem, we co-cultured a wild-type EC-H with an EC-H generated from calretinin-deficient mice. In these preparations only the mossy cell axons originating from the wild-type mouse contain calretinin and, thus, calretinin could be employed to visualize and quantify sprouting of associational and the commissural mossy cell axon collaterals, respectively.

Since we used tissue from calretinin-deficient mutant mice as a tool, we wondered whether removing calretinin from mossy cells could affect the sprouting response of the wild-type axons we were monitoring. Our observations do not provide any evidence for such an influence on wild-type sprouting axons since (i) sprouting in the wild-type-calretinin-deficient double cultures was similar to sprouting observed in the wild-type condition (associational sprouting), (ii) sprouting was not altered in the environment of the calretinin-deficient DG, and, (iii) sprouting was similar in virally traced wild-type and calretinin-deficient preparations. We conclude that although absence of calretinin from adult neurons may have some specific effects (Schurmans et al., 1997; Gurden et al., 1998), it does not seem to grossly affect the sprouting response of calretinin-positive wild-type axons in the hybrid EC-H double cultures.

### Associational and Commissural Axon Collaterals of Mossy Cells React Rapidly, Simultaneously and Massively to Entorhinal Denervation

Mossy cells are remarkable relay cells that connect large parts of both hippocampi (Scharfman and Schwartzkroin, 1988; Sloviter, 1994; Deller et al., 1995b; Zappone and Sloviter, 2001; Scharfman and Myers, 2012; Scharfman, 2016; Bui et al., 2018). Loss of hilar mossy cells is commonly seen in human temporal lobe epilepsy (Sloviter, 1996) and it can be reproducibly induced in temporal-lobe epilepsy mouse models (Kienzler et al., 2006, 2009; Volz et al., 2011; Kienzler-Norwood et al., 2017). Accordingly, mossy cells have received much attention in recent years and their role in physiology and pathology has been intensely investigated (see Scharfman, 2016 for review). In comparison, much less is known about their restorative abilities, e.g., their ability to reshape their axonal arbors using collateral sprouting. In particular in disease contexts, e.g., temporal lobe epilepsy, knowledge about their ability to contribute to brain repair could be helpful.

In the present study, we have seen that mossy cells react to denervation rapidly and very strongly, i.e., they start re-innervating the denervated DG within the first days after the lesion and they cover the entire denervated zone. Of note, both axon collaterals showed this massive growth response, suggesting that a given mossy cell can react with its entire axonal arbor to denervation. Furthermore, the sprouting response on one side did not appear to be limited by the fact that sprouting occurred on the other side. This massive growth propensity of the mossy cells is remarkable, since axonal sprouting – other than axonal regeneration following axotomy – does not require reprograming of the reacting neuron (Steward, 1995; Caroni, 1997). The ability of neurons to undergo this reprograming and to regenerate their axons is age-dependent and ends in mouse EC-H cultures after the first week *in vitro* (Prang et al., 2001, 2003; Del Turco and Deller, 2007). Mossy cells are, thus, using their structural plasticity programs to extensively re-innervate the dentate gyrus between DIV 14 and DIV 28.

Of note, the reactive response of mossy cell axons to entorhinal denervation is limited to the molecular layer of the dentate gyrus. Although entorhinal cortex lesion also denervates the neighboring stratum lacunosum-moleculare of CA3 and CA1, mossy cell axons stay within the DG and do not leave their home region. Since axonal guidance occurs at the growth cone of a growing axon (Campbell and Holt, 2001), a number of attractive or repulsive factors, for example matrix molecules (Smith et al., 2015), neurotransmitters and soluble neurotrophic factors (Zheng et al., 1994; Zhang and Poo, 2002) derived from neurons or glial cells, could play a role in this region-specific response (Goldberg, 2003; Stoeckli, 2018).

### Sprouting of Mossy Cell Axons *in vitro* Is Similar to Sprouting of Mossy Cell Axons in Juvenile Rodents *in viv*o

The sprouting response of mossy cell axons in our culture preparations was extensive and covered the entire denervated zone. This is much more extensive than what we have seen following entorhinal denervation in adult mice *in vivo* using a combination of anterograde tracing and calretinin-labeling (Del Turco et al., 2003). In adult brains calretinin-positive mossy cell axons grow only for short distances into the “entorhinal zone”. Nevertheless, the mossy cell sprouting response observed in adult mice was still more extensive than the one observed in rats, where mossy cell axons remain primarily within their home territory (Frotscher et al., 1997).

What could explain these differences in the sprouting propensity of mossy axons? With regard to the difference between the *in vitro* situation studied here and the sprouting observed in adult mice (Del Turco et al., 2003), we propose that the age of the cultures plays an important role. Although relevant maturation and differentiation steps have occurred (Prang et al., 2001, 2003), organotypic tissue cultures are still “juvenile” and, thus, highly plastic. More specifically, cultures are generated between postnatal days 4–5 and lesions are performed at DIV 14, corresponding to juvenile animals lesioned *in vivo* between 2–3 weeks postnatally. Such animals have been investigated previously and a much stronger sprouting response of juveniles has been reported compared to adults (Gall and Lynch, 1980, 1981; Gall et al., 1980): Mossy cell axons of juveniles leave their home layer in large numbers and re-innervate the entire denervated “entorhinal zone” of the dentate, similar to what we have seen *in vitro*. This effect is also much faster than in adults (Gall and Lynch, 1978; McWilliams and Lynch, 1983). Of note, strength as well as speed of re-innervation diminish with age (Gall and Lynch, 1981; Hoff et al., 1982a, b; McWilliams and Lynch, 1983), suggesting that the growth propensity or plasticity of mossy cells decreases with maturation (Caroni, 1997; Goldberg, 2003) or other molecules are expressed that restrict and pattern the sprouting response (Deller et al., 2000). The same considerations hold true for the differences observed between mouse and rat (Deller et al., 2007): Intrinsic factors regulating the strength of the growth response or extrinsic factors guiding and/or limiting the sprouting response of mossy cell axons could be different, which could explain the differences in mossy axon sprouting seen between the species.

## Data Availability Statement

Datasets generated for this study may be made available on request to the corresponding author.

## Author Contributions

DD, MP, VB, and LH-E acquired and analyzed the data. DD and TD conceived and supervised the study, and wrote the manuscript. All authors were involved in data interpretation, critically revising the manuscript, read, and approved the final manuscript.

## Conflict of Interest

The authors declare that the research was conducted in the absence of any commercial or financial relationships that could be construed as a potential conflict of interest.
